# Antimicrobial Clothing Based on Electrospun Fibers with ZnO Nanoparticles

**DOI:** 10.3390/ijms24021629

**Published:** 2023-01-13

**Authors:** Manuela Daniela Preda, Maria Leila Popa, Ionela Andreea Neacșu, Alexandru Mihai Grumezescu, Octav Ginghină

**Affiliations:** 1 Department of Science and Engineering of Oxide Materials and Nanomaterials, Faculty of Chemical Engineering and Biotechnologies, University Politehnica of Bucharest, 011061 Bucharest, Romania; 2 National Research Center for Micro and Nanomaterials, Faculty of Chemical Engineering and Biotechnologies, University Politehnica of Bucharest, 060042 Bucharest, Romania; 3 Research Institute of the University of Bucharest—ICUB, University of Bucharest, 050657 Bucharest, Romania; 4 Academy of Romanian Scientists, Ilfov No. 3, 050044 Bucharest, Romania; 5 Faculty of Medicine, University of Medicine and Pharmacy Carol Davila from Bucharest, 37 Dionisie Lupu Street, District 2, 020021 Bucharest, Romania

**Keywords:** electrospinning, zinc oxide, nanoparticles

## Abstract

There has been a surge in interest in developing protective textiles and clothes to protect wearers from risks such as chemical, biological, heat, UV, pollution, and other environmental factors. Traditional protective textiles have strong water resistance but lack breathability and have a limited capacity to remove water vapor and moisture. Electrospun fibers and membranes have shown enormous promise in developing protective materials and garments. Textiles made up of electrospun fibers and membranes can provide thermal comfort and protection against a wide range of environmental threats. Because of their multifunctional properties, such as semi-conductivity, ultraviolet absorption, optical transparency, and photoluminescence, their low toxicity, biodegradability, low cost, and versatility in achieving diverse shapes, ZnO-based nanomaterials are a subject of increasing interest in the current review. The growing uses of electrospinning in the development of breathable and protective textiles are highlighted in this review.

## 1. Introduction

### 1.1. Electrospinning

**Figure 1:**
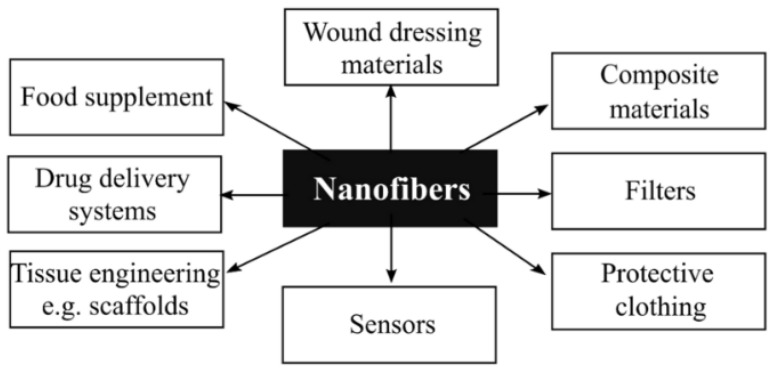
Applications of nanofibers in numerous aspects of daily life reproduced from [11].

Electrospinning creates long nanofibers with diameters ranging from 10 to 100 nm from various materials [1,2]. The first patent was granted in 1934 when Anton Formhals effectively spun cellulose acetate fibers from an acetone solution [3]. Meanwhile, the surge of interest in nanotechnology over the last two decades has rekindled interest in electrospinning in a variety of fields, such as healthcare [4], electronics [5], sensors [6], and fuel cells (Figure 1) [7].

This technology allows for the production of large-scale continuous nanofibers that can be tailored by altering the process parameters [2,8]. Because of its morphological and mechanical features, the electrospinning technology allows the fabrication of materials with regulated porosity and a large surface-to-volume ratio, which creates an interconnected network appropriate for biological applications like wound dressings [9], tissue engineering [10], and other cell growth applications due to the excellent biocompatibility and low toxicity.

Even though electrospinning is primarily used to make polymeric fibers, since it requires polymer solutions or melts, it can also be used to prepare mixtures of polymers with other materials. For example, two examples are embedding metallic or semi-conducting nanoparticles or producing fibers from a chemical solution. This enables the thorough mixing of polymeric and non-metallic components, as well as calcining the polymeric part after electrospinning to keep pure ceramic or other non-metallic materials. Compared to pure polymer nanofiber mats, such approaches may greatly expand the range of conceivable applications [12].

**Figure 2:**
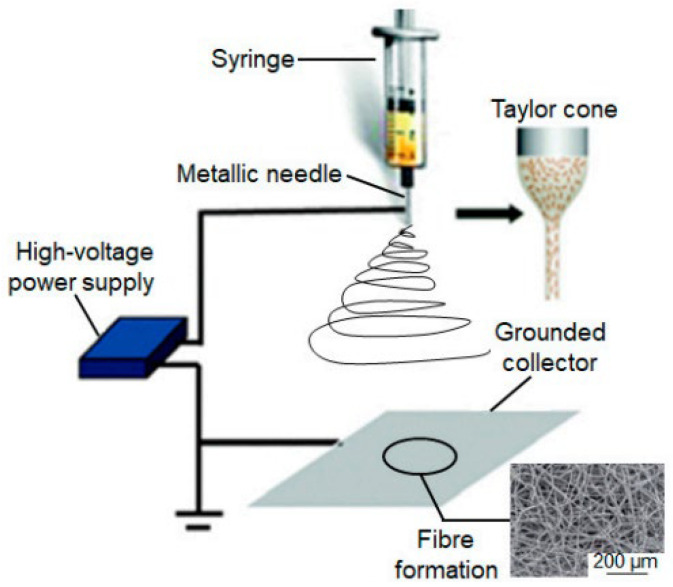
The main electrospinning mechanism and equipment. The usual electrospinning setup is depicted schematically in this diagram, reproduced from [14].

Electrospinning appears to be simple to use at first look. A common setup consists of four major components: a pump, a syringe coupled to a small-diameter needle, a high-voltage source, and a grounded metal collector. During the procedure, an electric field is applied to a polymer solution, and the voltage is raised until the electrical force is sufficient to overcome the solution’s surface tension. The solvent evaporates as a jet of solution (emitted by a Taylor cone) is directed into the collector (Figure 2). Finally, the polymer is gathered into a mesh. To be electro-spinnable, the polymer solution must be above a certain concentration (for chain entanglement) and operated under appropriate process parameters such as applied voltage, flow rate, and needle-to-collector distance. Finding a good set of settings to electrospin defect-free fibers can be difficult because of these variables. As a result, extensive research has been undertaken into the electrospinning of various polymers under various operating circumstances [3,13].

### 1.2. Zinc Oxide

Zinc oxide (ZnO) has been increasingly popular in material science in recent decades due to its multifunctional features [15], inexpensive cost [16], and wide range of uses in numerous research fields and applications [17]. The boost in scientific interest was mirrored by significant growth in the ZnO market in the industry, with applications in rubber [18], ceramic materials [19], paints [20], food packaging [21], cosmetics [22], and pharmaceutical items [23,24].

Furthermore, ZnO is regarded as a bio-safe material, and the Food and Drug Administration (FDA) has allowed its usage in cosmetic items, which is undoubtedly a driving force in ZnO market expansion [25]. In nanostructured form (i.e., forms having at least one characteristic dimension smaller than 100 nm) [26], ZnO becomes even more intriguing, allowing the creation of new nanomaterials [27] and nanodevices [28] with unique chemical-physical characteristics [29]. Furthermore, it has the advantage of being simple to synthesize using a variety of techniques to produce a diverse range of nanostructures (NStr), such as nanoparticles (NPs) [30], nanowires (NWs) [31], nanofibers (NFs) [32], nanoflowers (NFls) [33], nanorods (NRs) [34], nanosheets (NSs) [35], nanotubes (NTs) [36], nanoribbons (NRBs) [37], and tetrapods (TPs) [38]. Nanostructured ZnO is increasingly being used in biomedical and healthcare domains, too [39,40], for antibacterial materials [41], tissue-engineering scaffolds [42], wound healing [43], drug delivery [44], molecular biosensors [45], fluorescence imaging [46] applications, among others.

Because ZnO nanostructures may form reactive oxygen species (ROS) and release Zn^2+^ ions, they are physiologically active, and toxicological studies always accompany the creation of novel ZnO-based nanomaterial to assess their biocompatibility [47]. While the use of modest doses of ZnO NStr enhances cell development, differentiation, and proliferation [48], as well as tissue regeneration, enhancing angiogenesis and osteointegration processes [49], multiple studies have shown that this is accompanied by ZnO antibacterial and antifungal capabilities [50]. Furthermore, ZnO NStr exhibits selectivity for specific cell lines, making them prospective candidates for cancer cell killing [51].

Aside from toxicological concerns, the relationship between ZnO-based systems (materials and nanocomposites) and biological microenvironments is particularly important for a wide range of target applications, requiring significant effort during the materials design and manufacturing stages to ensure safe and effective application.

### 1.3. Electrospun Fibers

It is commonly recognized that when materials are designed to the nanoscale, they can acquire unique features that can potentially have far-reaching consequences in various fields. The specific surface area, for example, is inversely proportional to the size—the specific surface area of a fiber increases as its diameter decreases [52]. As the diameter of the fiber decreases from 500 nm to 5 nm, the surface area per kilogram of fiber increases considerably, from roughly 10,000 to 1,000,000 m^2^/kg [53]. The fiber at the nanoscale allows the appropriate fibrous structure to achieve a high porosity of up to 99% [54].

Electrospun fibers have a very high specific surface area, which allows them to have a very high capacity and a large number of adsorption sites for efficiently capturing or releasing particles, molecules, and functional groups, among other things [55,56]. This capacity can improve sensitivity and responsiveness [57] by allowing it to interact closely with surrounding materials and detect the external world more efficiently.

Furthermore, the electrospun nanofibers might be additionally formed into an organized structure, such as bundles [58] or skeins [59], to boost their practical use and applications. The distinctive qualities of bundles [58] and yarns [59], such as flexibility and usefulness, in which the number of electrospun nanofibers in a unit area of cross-section is greatly enhanced, would be improved and highlighted when compared to old-fashioned yarns. Synthetic fibers, for example, are well recognized in material science for their inability to have both high toughness and high strength [60]. Electrospun fibrous fibers with toughness and strength equivalent to spider silk were used in a recent study to overcome this inherent contradiction. This extraordinary yarn was created by combining poly (acrylonitrile-co-methyl acrylate) with a small amount of poly (ethylene glycol) bisazide as a crosslinker, then annealing under tension. The spider silk-like qualities of the resultant cross-linked aligned multi-fibrillar yarns include a tensile strength of almost 1.3 GPa and a toughness of around 137 J/g [61].

In summary, tailoring structures at multiscale and selecting polymers whose properties are determined by polymer architecture, which is controlled by macromolecular design and processing, makes it possible and appealing to generate electrospun fibers with excellent mechanical properties, from single fibers to bundles and yarns [58,59,62,63].

### 1.4. Protective Clothing

Protective clothing is described as textile constructions that shield the human body from external threats [64], such as a bullet, biological or chemical agents [65], fire, cold, or heat [66]. In general, the main purpose of protective clothing development is to balance the competing goals of generating low-cost, lightweight, and comfortable clothing systems that function well [67].

Clothes comfort as a psychological experience is a significant feature of protective clothing, resulting from integrated cognitive, visual, tactile sensory, body-clothing interactions, and environmental factors. Thermal, mechanical, psychological, and sensory comfort are components of clothing comfort [64].

There are several ways to immobilize bioactive substances on textile fibers, each with its advantages and disadvantages. The fabric is first treated and functionalized to enhance the impregnation of the chosen bioactive substances and their durability within the textile [68].

There are several techniques for trapping bioactive agents onto textile fibers, each with its own set of specifications, benefits, and drawbacks, with the fabric being earlier treated and functionalized to improve the impregnation of the chosen bioactive agents as well as their longevity within the fabric [68]—however, the most recent studies use the electrospinning method to obtain textiles with antimicrobial properties.

## 2. Design of Antimicrobial Composite Fibers Based on Electrospun Polymers and Inorganic Nanoparticles

Electrospinning and similar methods have been used to integrate many inorganic nanostructures into polymeric fibers [1]. Examining well-known scientific data sources yields intriguing statistical insights. Over the previous decade, about 4000 publications on nanofibrous composites have been published. Approximately 30% of these papers focused on the application of well-known antibacterial metallic (silver [69] and gold [70]) and metal oxide nanoparticles (mostly those based on zinc [71], titanium, copper [72], and iron [73]).

The growing interest in metal oxide nanoparticles stems mostly from their easy and low-cost synthesis, which is often accomplished using solution-phase processes (sol-gel [74], co-precipitation [75], hydrothermal [76], etc.) and allows for great control of particle form. Furthermore, such nanoparticles are generally eco-friendly. Metal oxides, particularly those based on zinc [77], iron [78], titanium [79], and copper [80], have also been explored as antibacterial species when integrated into (bio)polymeric fibers.

Polymeric fibers derived from electrospinning techniques are ideal for applications in tissue engineering (scaffolds and/or wound healing materials) due to the sub-micrometric fibrous structure with interconnected pores that mimic the extracellular matrix, promoting the adhesion and proliferation of distinct tissue cells, as mentioned in the Introduction. Nonetheless, that shape is prone to harmful microbe attachment, which can form a protective biofilm (composed of extracellular DNA, proteins, and polysaccharides) [81]. As a result, novel electrospun composite materials with antibacterial characteristics are becoming increasingly popular.

Although various organic compounds have antibacterial action, nanoparticles have lately been chosen because they can bestow new capabilities and/or improve existing ones. As a result, the next section will focus on electrospinning and associated approaches for designing composite fibers and then on the most researched antimicrobial nanoparticles and their influence on the creation and final characteristics of (bio)polymeric fibers.

## 3. Approaches for Designing Fibrous Composite Materials by Electrospinning and Related Techniques

**Figure 3:**
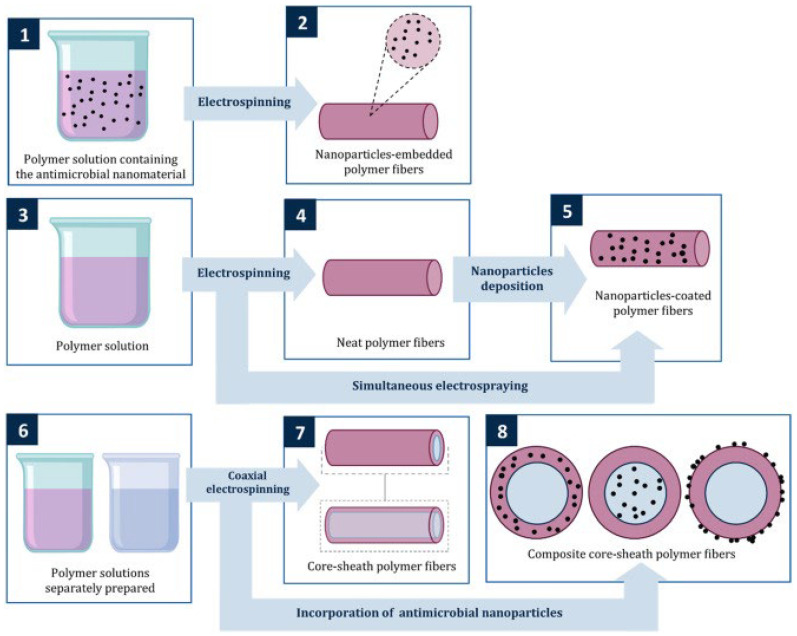
Scheme of methodologies for constructing composite fiber materials manufactured by electrospinning and related processes, as well as the corresponding outer and inner morphologies: (1–2) simple electrospinning, (3–5) electrospinning/electrospraying, and (6–8) coaxial electrospinning [84]. Reprinted from Materials Science and Engineering: C, Vol 101, Heriberto Rodríguez-Tobías, Graciela Morales, Daniel Grande, Comprehensive review on electrospinning techniques as versatile approaches toward antimicrobial biopolymeric composite fibers, 306–322, Copyright (2019), with permission from Elsevier.

The most often described method for producing composite fibers is simple electrospinning, which involves creating a polymeric solution incorporating antimicrobial nanoparticles or precursory chemicals, which is then electrospun (Figure 3; 1–2). For example, Hui Wu et al. used this method in their research and obtained ultra-thin zinc oxide fibers. Sol-gel processing and electrospinning were used with poly(vinyl acetate) and zinc acetate as precursors [82]. Another example is the case where Beregoi et al. used three simple approaches (electrospinning, sol-gel synthesis, and electrodeposition) to create core-double shell nylon-ZnO/polypyrrole electrospun nanofibers [83].

The second strategy is electrospinning/electrospraying, which involves simultaneous electrospinning of a polymeric solution and electrospraying of the matching antimicrobial nanoparticles dispersion (Figure 3; 3–5) [84]. These two electro-hydrodynamic techniques were used in their research by Grande et al. for the development of poly(3-hydroxybutyrate) (PHB)-based nanofibrous hybrid materials containing zinc oxide nanoparticles (nano-ZnO). They electrospinning the polymer/nano-ZnO solutions and the combination of electrospinning of polymer solutions and electrospraying of nano-ZnO dispersions [85]. A more recent study where researchers used these two integrated techniques is reported by Heriberto Rodríguez-Tobías et al., where, for the first time, an exhaustive examination of the photo-degradation behavior of ZnO-embedded and ZnO-coated PHB (poly(3-hydroxyalkanoate)) mats generated by electrospinning and electrospinning/ electrospraying procedures was conducted [86].

Another noteworthy approach is coaxial electrospinning, which may be done in a similar setup to standard electrospinning. Still, the ejection device comprises concentric needles, which gather core-sheath fibers as solutions are ejected from them [87]. Combining this approach with others linked to electrospinning might result in a wide range of morphologies employed as antimicrobial devices for medical and environmental problems (Figure 3; 6–8) [88]. For this case, as an example, Rungthiwa Methaapanon et al. used this method to obtain ZnO/PAN (polyacrylonitrile) nanofibers [89]. Table 1 presents several advantages and disadvantages of different approaches for designing fibrous composite materials by electrospinning.

## 4. Antimicrobial Nanoparticles and Their Influence on the Creation and Final Characteristics of (Bio)Polymeric Fibers

The development of antimicrobial protective clothing involves the fabrication of cost-effective antimicrobial materials. The most researched antimicrobial nanoparticles include TiO_2_ [91,92], ZnO [93,94], MgO [95], and CaO [96,97]. However, to create antibacterial nanofibers, ZnO and electrospinning technology have frequently been coupled in studies.

In 2019, for the first time, Abdul WahabJatoi et al. reported the synthesis of ZnO/AgNPs by a facile solution mixing procedure, fabrications of CA/ZnO/AgNPs, and characterization of CA/ZnO/AgNPs for sustained antibacterial activities. In the current study, ZnO/AgNPs were created for the first time utilizing a straightforward solution mixing process with Dopa acting as both a powerful adhesive (prevents the negative consequences of the silver release) and a reducing agent [98].

**Figure 4:**
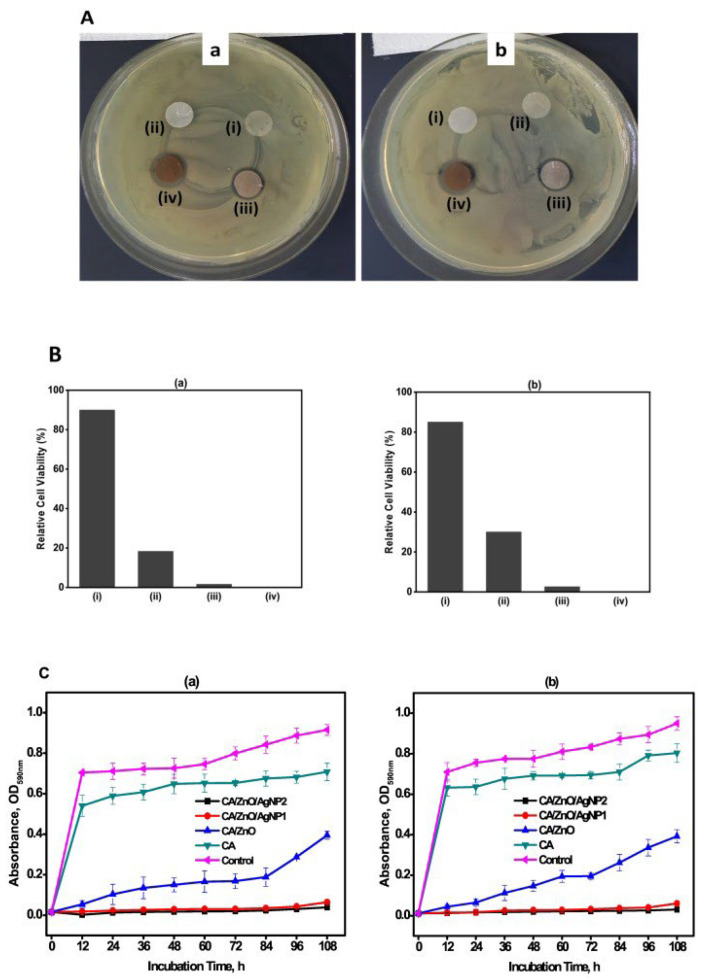
Antibacterial activity results. (**A**) Photographic images of disc diffusion test. (**B**) Bactericidal assay. (**C**) Liquid medium bacteria growth inhibition of the samples (i) CA, (ii) CA/ZnO, (iii) CA/ZnO/AgNP1, and (iv) CA/ZnO/AgNP2 against (**a**) *E. coli* and (**b**) *S. aureus strains* [98]. Reprinted from Materials Science and Engineering: C, Vol 105, Abdul Wahab Jatoi, Ick Soo Kim, Hiroshi Ogasawara, Qing-Qing Ni, Characterizations and application of CA/ZnO/AgNP composite nanofibers for sustained antibacterial properties, 110077, Copyright (2019), with permission from Elsevier.

The agar plate disc diffusion (Figure 4A) test revealed that the CA/ZnO/AgNPs samples effectively restrict the growth of both strains. The quantitative bactericidal test findings shown in Figure 4B demonstrated that the CA/ZnO/AgNP2 samples had 100% bactericidal characteristics (0% viable cells) against both test strains. CA/ZnO/AgNP1 showed 1.6% and 2.8% cell viability in *Escherichia coli (E. coli)* and *Staphylococcus aureus (S. aureus)* strains, respectively. These findings point to CA/ZnO/AgNP2 as a promising antibacterial nanocomposite. To examine the growth inhibition capabilities of the samples in a liquid medium, growth curves of *E. coli* and *S. aureus* strains incubated with samples were obtained. The results shown in Figure 4C show that CA/ZnO/AgNPs samples effectively reduced the development of both bacteria for up to 108 h (tested time) [98].

The use of ZnO nanorods and Ag nanoparticles-decorated multifunctional electrospun poly(methyl methacrylate) (PMMA) nanofibers (PMMA/ZnOAg NFs) in protective mats are being investigated in this study. PMMA/ZnOAg NFs with an average diameter of 450 nm were directly electrospun on a non-woven fabric from mixtures containing PMMA, ZnO nanorods, and Ag nanoparticles. PMMA, ZnO nanorods, and Ag nanoparticles are present [99].

**Figure 5:**
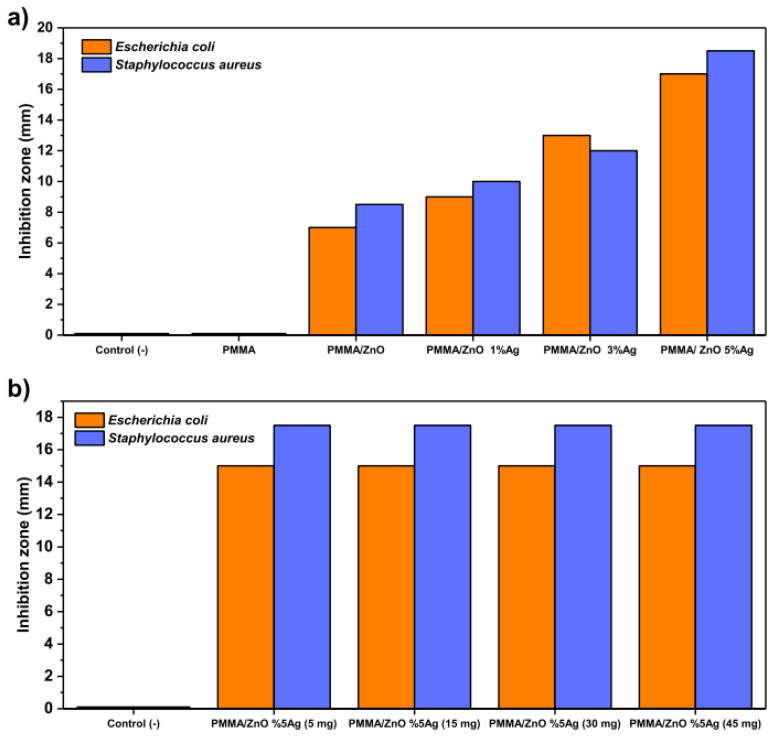
Antibacterial activity of NF mats. (**a**) Antibacterial activity of different types and different Ag concentrations of NF mats. (**b**) Antibacterial activity of different amounts of PMMA/ZnO-5% Ag NF mats [99]. Reprinted with permission from ACS Appl. Mater. Interfaces 2021, 13, 4, 5678–5690. Copyright (2021) American Chemical Society.

The presence or absence of inhibitory zones is used to assess qualitatively. According to the agar diffusion method results (Figure 5a), *S. aureus* was the most vulnerable microbe that PMMA/ ZnOAg NFs strongly suppressed. The effect of the amount of PMMA/ZnOAg NFs on the antibacterial action was also investigated. In comparison to the previously stated investigation, the results demonstrated that the antibacterial activity did not alter significantly with varied dosages (5, 15, 30, and 45 mg) of the substance (Figure 5b) [99].

**Figure 6:**
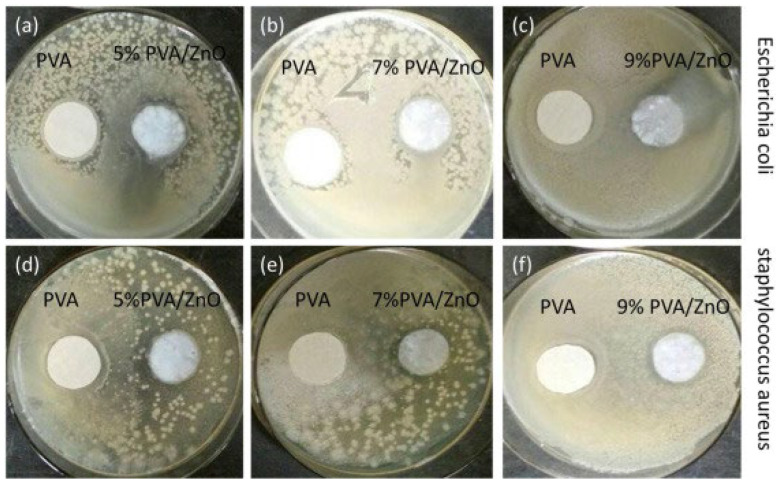
Antibacterial results of PVA nanofibers and PVA/ZnO nanofibers [100].

Khan et al. effectively combined PVA/ZnO using electrospinning to create the multifunctional nanocomposite. ZnO nanoparticles were mixed with PVA at three distinct concentrations—5 wt. %, 7 wt. %, and 9 wt. %—to produce homogeneous dispersion on nanofibers. The agar diffusion plate method was utilized to investigate the antibacterial characteristics of the PVA/ZnO nanofibers. *Staphylococcus aureus* and *Escherichia coli* were utilized as model microorganisms in this procedure. Bacteria were applied to the cool PVA nanofibers and PVA/ZnO nanofibers. The results showed that ZnO nanoparticle-treated nanofibers exhibit significant antibacterial action against *S. aureus* and *E. coli* germs. The killing process of bacteria was accelerated as the concentration of ZnO nanoparticles was raised. As demonstrated in Figure 6, plain PVA has no antibacterial activities, while 9 wt. % PVA/ZnO nanofibers membranes have the strongest antibacterial properties against *Staphylococcus aureus* [100].

According to health research, reinforcing gelatin is required to obtain the multifunctional substance. In this study, nano zinc oxide (ZnO; concentrations of 0.5%, 1%, and 1.5%) was doped with gelatin and electrospun under certain conditions to produce multifunctional gelatin/ZnO nanofibers [101]. 

**Figure 7:**
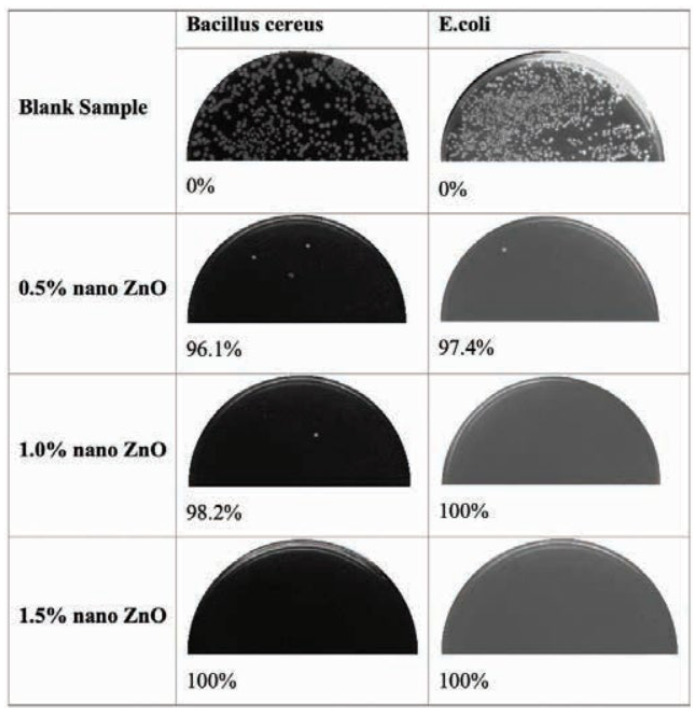
Antibacterial efficiency of blank and treated samples [101].

Figure 7 depicts the outcomes of the antibacterial experiment. As demonstrated, the blank sample has no antibacterial ability against Gram-negative and positive bacteria. However, the samples containing micro ZnO have antibacterial properties. As shown in Figure 7, the sample containing 1.5% nano ZnO has 100% antibacterial activity against both bacteria. In comparison, the antibacterial activity of the sample treated with 1% nano ZnO is approximately 100% and 98.2% for *E. coli* and *Bacillus cereus*, respectively, and 97.4% and 96.1% for the sample containing 0.5% nano ZnO. When the antibacterial properties of samples are compared, it is discovered that the antibacterial properties of samples against *E. coli* are greater than those of *Bacillus cereus* due to differences in cell wall thicknesses [101].

In another study, electrospun nanofibers were created with different quantities of aloe vera (1%, 2%, 3%, 4%) while maintaining consistent amounts of ZnO NPs (0.5%).

Due to the lack of mobility of ZnO NPs from fibers to the external media, a qualitative antibacterial investigation revealed that samples containing various concentrations of ZnO NPs did not demonstrate any zone of inhibition. When samples with varying concentrations of ZnO NPs were compared to samples with variations in aloe vera, the quantitative antibacterial analysis produced outstanding results. The explanation is that aloe vera is consumed while killing the germs, whereas ZnO NPs remain lodged in the nanofiber structure and exhibit long-term antibacterial activity. Furthermore, ZnO NPs are semiconductors, and their redox processes destroy germs more efficiently than real aloe vera, which lacks this feature (Table 2) [102].

When Norouzi et al. prepared ZnO NPs(C) using zinc acetate dehydrate and NaOH, they discovered that the fibers they electrospun from ZnO NPs and polyvinyl alcohol had antibacterial activity against *S. aureus* and *E. coli*.

The minimum inhibitory concentration (MIC) and minimum bactericidal concentration (MBC) average values indicating the minimum concentrations of antibacterial nanoparticles (zinc oxide) were measured to ensure the antibacterial efficiencies of PVA/ZnO composite nanofibers against *S. aureus* and *E. coli*. Table 3 shows that all values are less than 500 g/mL. Thus, according to the cytotoxicity results, the ZnO nanoparticles were toxic to the bacterium at these concentrations. In contrast, the pure PVA nanofibers showed no antimicrobial effect, confirming the role of zinc oxide nanoparticles in antibacterial activities. Ampicillin indicated the most effective antibacterial activities against *E. coli*; however, ZnO nanoparticles displayed more effective antimicrobial activity against *S. aureus* [103].

Other researchers used electrospinning to create polyacrylonitrile (PAN) polymer nanofibers. The surface of the electrospun PAN nanofibers membrane was functionalized with ZnO-Ag heterostructure nanoparticles via three different chemical pathways, such as reflux, blending, and hydrothermal methods, and the resulting composite nanofibers membranes were designated as PAN/ZnO-Ag (R), PAN/ZnO-Ag (B), and PAN/ZnO-Ag (H) [104].

**Figure 8:**
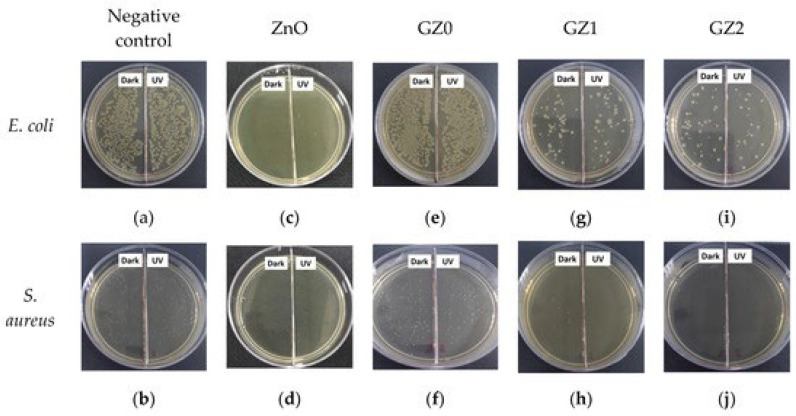
The photographs show the antibacterial activity of (**a**,**b**) negative control, (**c**,**d**) ZnO particles, (**e**,**f**) GZ0, (**g**,**h**) GZ1, and (**i**,**j**) GZ2 against *S. aureus* and *E. coli*, respectively [106].

The PAN-ZnO nanofibers membrane exhibits good antibacterial activities against gram-positive *M. luteus* but is unable to prevent the growth of gram-negative *E. coli* bacteria (Figure 8b,e). Similarly, PAN-Ag nanofiber membranes inhibited growth (Figure 8a,d), and the data demonstrated strong antibacterial activity against both *M. luteus* and *E. coli* bacteria [104].

In a recent study, Permyakova et al. created ZnO-modified polycaprolactone nanofibers (PCL-ZO) by plasma treating the nanofiber surface in a gaseous combination of Ar/CO_2_/C_2_H_4_, followed by ZnO nanoparticle deposition (NPs). The mats displayed strong antibacterial and antifungal activity when tested against Gram-positive and Gram-negative pathogenic bacteria and harmful fungi [105].

**Figure 9:**
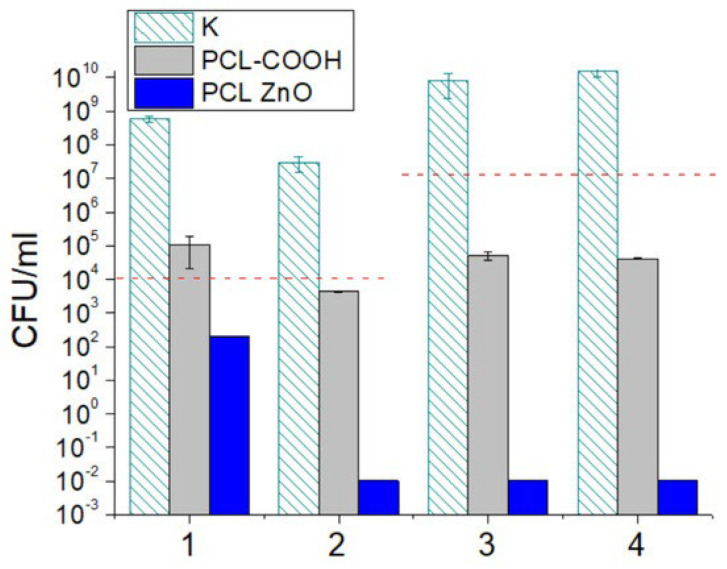
Antifungal and antibacterial activities of PCL-COOH and PCL-ZnO against *Candida parapsilosis* ATCC90018 (1), *Neurospora crassa* (2), *Escherichia coli* U20 (3), and *Staphylococcus aureus* CSA154 (4) assessed by CFU count after 24 h. K—control without sample. Dashed horizontal lines show initial cell concentrations [105].

The number of CFUs after 24 h was used to assess the antibacterial and antifungal properties of PCL-ref and PCL-ZnO (Figure 9). The number of CFUs increased by 3–4 orders of magnitude in the control sample. PCL-ref showed significant antibacterial activity compared to the control well without a sample (K) (approximately 2-log reduction in CFUs). In fungal cultures, CFUs grew slightly (*Candida parapsilosis*) or declined (*Neurospora crassa*). The PCL-ZnO sample significantly reduced *Candida parapsilosis* (blue column (1), CFUs and had 100% antibacterial/antifungal activity against *Neurospora crassa* (2), *E. coli* (3), and *S. aureus* (4) strains [105].

Yu Chen and co-workers fabricated gelatin/ZnO fibers via a side-by-side electrospinning technique as a potential wound dressing. The viable colony count method was used to identify the bacterium. Photographs in Figure 9 depict the antibacterial activity of gelatin/ZnO fibers against *S. aureus* and *E. coli*. Pure gelatin fibers (GZ0) show no harmful effect on bacterial strains, whereas pure ZnO, GZ1, and GZ2 have outstanding bacteriostatic properties. Furthermore, GZ2 with a higher concentration of ZnO exhibits stronger antibacterial action than GZ1 with a low concentration of ZnO.

The superoxide radicals (O_2_) created by ZnO particles, per the documented literature, are what give gelatin/ZnO fibers their antibacterial properties. The bacterial cell wall can be attacked by superoxide radicals, causing cell wall leakage and bacterial death [47].

**Figure 10:**
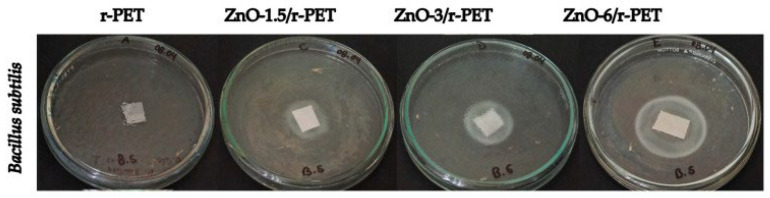
Antibacterial tests *Bacillus subtilis* in polymer composite fibers [107].

**Figure 11:**
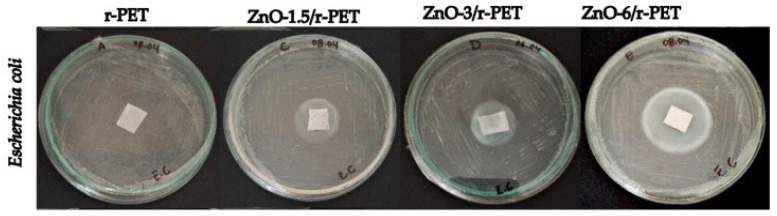
Antibacterial tests of *Escherichia coli* in r-PET composite fibers [107].

This study developed polymeric fibers made of recycled polyethylene terephthalate (r-PET) from post-consumer water bottles to assess their antibacterial and antifungal properties. These fibers were functionalized with 0%, 1.5%, 3%, and 6% zinc oxide nanoparticles (ZnO-NPs) in the function of r-PET weight. The fibers were created by electrospinning and had a diameter range of 200–5000 nm, whereas the ZnO–NPs were created using a solvothermal technique, yielding particles with a mean diameter of 38.15 nm. Using the agar diffusion method, the functionalized fibers were tested against Bacillus subtilis (Figure 10) and Escherichia coli (Figure 11). The maximum inhibitory halo was obtained at 6% weight-averaged ZnO-NPs, measuring 26.5 mm and 34.25 mm, respectively [107].

A number of other polymers have also been investigated as potential antibacterial substances. For example, a group of researchers introduced Curcumin and ZnO to the PCL/CS nanofibrous simultaneously for the first time. They obtained five samples (PCL15-pure PCL 15 wt%; PCL15CS3-PCL 15 wt% CS 3 wt%; PCL15CS3ZnO1-PCL 15 wt% CS 3 wt% ZnO 1 wt%; PCL15CS3ZnO1Cur1-PCL 15 wt% CS 3 wt% ZnO 1 wt% Cur 1 wt%; and PCL15CS3ZnO1Cur3-PCL 15 wt% CS 3 wt% ZnO 1 wt% Cur 3 wt%) [108].

**Figure 12:**
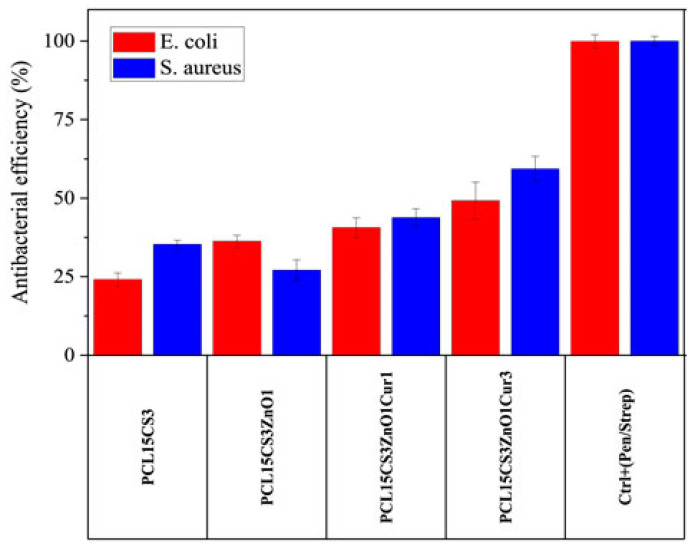
Antibacterial efficiency of the electrospun samples against *E. coli* and *S. aureus* bacteria after 24 h [108].

Electrospun nanofibers were tested for antibacterial efficacy against two species of bacteria, *Escherichia coli* (*E. coli*) and *Staphylococcus aureus* (*S. aureus*) (Figure 12). Pure PCL15 showed no inhibition, as expected. The results showed that including CS in the PCL scaffold increased antibacterial efficacy against both microorganisms. The antibacterial effectiveness of the PCL15CS3 scaffold against *E. coli* and *S. aureus* was 24.1 2.1 and 35.3 1.3, respectively. This is due to the interaction of the CS NH2 groups with the PCL chains. Furthermore, CS is a natural antibacterial agent that is effective against bacteria and viruses, and the positive charge of chitosan interacts with the negatively charged cell membranes to kill germs [108].

Another group of researchers reports ZnO/PLGA/PCL nanofibers for antibacterial material. An oblique section free surface electrospinning (OSFSE) apparatus was used in this study to create high-quality and high-output ZnO/PLGA/PCL nanofiber [109].

The findings demonstrated that adding ZnO to nanofibers could significantly increase their antibacterial properties. The higher the ZnO concentration, the stronger the antibacterial potential of nanofiber [109].

The use of electrospinning to create ZnO-containing PVA fibers is a helpful proposition. In this study, freeze-dried ZnO powder and as-precipitated ZnO solution were produced using a unique flow synthesis process. For the fabrication of the electrospun fiber mats, two different concentrations of ZnO were utilized. PVA/ZnO electrospun fibrous mats were then created using varying amounts of ZnO. Antibacterial fibrous PVA/ZnO composites can be successfully manufactured via electrospinning, as demonstrated here. The antibacterial properties of the electrospun mats were investigated utilizing the zone inhibition method on Gram-positive *S. aureus* bacteria. The results showed that raising the concentration of ZnO increased the antibacterial capabilities [110].

## 5. Conclusions

Electrospinning, for example, can be used to distribute inorganic antibacterial particles and agents both within and on the surface of polymer fibers. When combined into garments, these fibers can provide antibacterial characteristics. Thus, electrospinning has enormous promise for developing membranes and fibers for protective clothing applications.

Furthermore, functional and smart fabrics can be created by mixing two distinct materials and electrospinning the composite fibers. These fibers may then be combined with a garment membrane to develop effective and practical materials. The fibers can be electrospun directly onto the fabric. Despite the fact that this method overcomes the challenges associated with seaming the electrospun membrane and fabric together, adhesion between the membrane and fabric remains problematic. Few research organizations are investigating plasma treatment and chemical additive strategies to improve membrane-fabric adhesion.

## Figures and Tables

**Table 1:** Advantages and disadvantages of different approaches for designing fibrous composite materials by electrospinning.

Type	Advantages	Disadvantages	Ref
Simple electrospinning	polymeric fibers with nanoparticles mainly incorporated (some particles could, however, migrate to the surface of the fibers during the electrospinning process)	limited antibacterial efficacy, which is attributable to the embedding of nanoparticles, which reduces the surface area susceptible to interaction with pathogen microorganisms	[84]
Electrospinning/electrospraying	of creating nanoparticle-coated polymeric fibers in a single step	additional equipment (such as electrospraying) is required for the manufacture of composite fibers, which raises the initial technological expenditure	[84]
Coaxial electrospinning	include the capacity of miscible and immiscible polymers to produce core-shell nanofibers, the high loading capacity of bioactive chemicals, continuous release from the fibers, and a less abrasive technique that makes it possible to transport vulnerable substances	A technique frequently yields fibers with various characteristics throughout the mesh layer	[90]

**Table 2:** Antimicrobial activity of all electrospun samples with variation in aloe vera and ZnO NPs against *S. aureus* and *E. coli* [102].

S. No	Percentage of Bacterial Reduction after 24 h	
	*S. aureus*	*E. coli*
10% PVA/1% aloe vera/0.5% ZnO NPS	75.5%	60.9%
10% PVA/2% aloe vera/0.5% ZnO NPS	79.1%	73.9%
10% PVA/3% aloe vera/0.5% ZnO NPS	86.1%	81.8%
10% PVA/4% aloe vera/0.5% ZnO NPS	91.2%	86.9%
10% PVA/0.5% aloe vera/1% ZnO NPs	93.50%	83.14%
10% PVA/0.5% aloe vera/2% ZnO NPs	97.20%	92.96%
10% PVA/0.5% aloe vera/3% ZnO NPs	99.80%	96.87%
10% PVA/0.5% aloe vera/4% ZnO NPs	100%	99.20%

**Table 3:** MIC and MBC of the PVA and PVA/ZnO composite nanofibers [103].

Sample	Strains			
	*E. coli* (ATCC:25922)		*S. aureus* (ATCC:6538)	
	MIC	MBC	MIC	MBC
PVA/ZnO	62.5 ± 00 μg/mL	125 ± 00 μg/mL	250 ± 00 μg/mL	250 ± 00 μg/mL
PVA	-	-	-	-
Ampicillin	4 ± 00 μg/mL	16 ± 00 μg/mL	250 ± 00 μg/mL	500 ± 00 μg/mL

## Data Availability

Not applicable.
